# Incidence rates of non-Hodgkin lymphoma subtypes among Black Africans with and without HIV in South Africa: a national registry-based study

**DOI:** 10.1016/j.lanafr.2026.100058

**Published:** 2026-05-19

**Authors:** Carole Metekoua, Yann Ruffieux, Tracey Wiggill, Tinashe Tombe-Nyahuma, Jenny Mary Mathew, Judith Mwansa-Kambafwile, Julia Bohlius, Meredith S. Shiels, Jun Tao, Mazvita Muchengeti, Eliane Rohner

**Affiliations:** a National Cancer Registry, National Institute of Communicable Diseases, National Health Laboratory Service, Johannesburg, South Africa; b Graduate School for Health Sciences, University of Bern, Bern, Switzerland; c Institute of Social and Preventive Medicine, University of Bern, Bern, Switzerland; d Division of Medical Microbiology and Immunology, Faculty of Health Sciences, University of Stellenbosch, Cape Town, South Africa; e Tygerberg Hospital, National Health Laboratory Service, Cape Town, South Africa; f Division of Epidemiology and Biostatistics, School of Public Health, University of Witwatersrand, Johannesburg, South Africa; g School of Public Health, University of Cape Town, Cape Town, South Africa; h Swiss Tropical and Public Health Institute, Allschwil, Switzerland; i University of Basel, Basel, Switzerland; j Division of Cancer Epidemiology and Genetics, National Cancer Institute, Rockville, USA; k South African DSI-NRF Centre of Excellence in Epidemiological Modelling and Analysis (SACEMA), Stellenbosch University, Stellenbosch, South Africa

**Keywords:** Non-Hodgkin lymphoma, HIV, South Africa, Black African

## Abstract

**Background:**

The impact of HIV on incident non-Hodgkin lymphoma (NHL) varies by subtype. We compared the incidence rates of NHL subtypes (aggressive and indolent) between Black Africans with and without HIV in South Africa from 2010 to 2021.

**Methods:**

We identified incident NHL among children and adults from the South African National Cancer Registry and stratified yearly population by HIV status, sex, age group and health care sector. We applied Poisson regression models, adjusting for sex, age, and calendar period, to estimate adjusted incidence rate ratios (aIRRs) for NHL and its subtypes among Black Africans with and without HIV. We calculated aIRR stratified by calendar period, sex, and age group.

**Findings:**

We identified 11,187 new NHL; 54% (n = 6023) occurred in male individuals. The risk of all NHL combined was higher among Black Africans with HIV than those without HIV, but the strength of the association varied by subtype. The aIRRs were highest for cutaneous T-cell lymphoma, diffuse large B-cell lymphoma and Burkitt lymphoma. There was no evidence that the strength of association changed over calendar period for most subtypes. The positive association with HIV was stronger among male individuals and those <45 years for all NHL combined and many subtypes.

**Interpretation:**

In South Africa, incidence rates of all NHL subtypes are higher among Black Africans with HIV than those without HIV. Further context specific studies are needed to clarify the underlying mechanisms such as immune dysfunction, chronic inflammation, or oncogenic co-infections to inform early NHL diagnosis and timely treatment.

**Funding:**

US National Institutes of Health, Swiss National Science Foundation and Swiss Cancer Research foundation.

## Introduction

People with HIV (PWH) continue to experience a higher risk of non-Hodgkin lymphoma (NHL) than the general population, despite the widespread availability of antiretroviral therapy (ART).^1^ This persistently increased risk could be attributed to incomplete immune reconstitution, HIV-induced chronic inflammation and immune activation, genetic alterations, elevated susceptibility to oncogenic viruses such as Epstein–Barr virus (EBV) or human herpesvirus 8, and premature cell aging as PWH live longer.^2–4^ The impact of HIV on NHL incidence varies across subtypes. Aggressive B-cell lymphomas like diffuse large B-cell lymphoma (DLBCL), Burkitt lymphoma (BL), plasmablastic lymphoma (PBL), primary effusion lymphoma (PEL), primary central nervous system lymphoma (PCNSL), and NHL not otherwise specified (NHL-NOS), show markedly elevated risks in PWH.^1,5–7^ Slightly increased risks among PWH have been observed in the United States (US) for marginal zone lymphoma (MZL), anaplastic large cell lymphoma (ALCL), peripheral T-cell lymphoma (PTCL) and precursor NHL, while other subtypes such as follicular lymphoma (FL) and chronic lymphocytic leukaemia/small lymphocytic lymphoma (CLL/SLL) showed no association with HIV.^5^ The relative risk of most NHL subtypes among PWH in the US declined between 2001 and 2019.^7^ South Africa has the world’s highest burden of HIV, with Black Africans disproportionately affected.^8^ The country’s ART programme started in 2004 and has undergone significant expansion. ART coverage in PWH exceeded 50% following the adoption of the universal test-and-treat policy in 2016 and reached an estimated 71% by 2021.^9^ During the ART era, studies in sub-Saharan Africa have mostly focused on the association between HIV and all NHL combined or aggressive B-cell NHL subtypes only.^6,10–12^ This focus partly reflects limitations in diagnostic capacity and the predominance of hospital-based studies, where indolent NHL subtypes are rarely captured.^1,6,11,12^ This is the first national-level study in South Africa comparing the incidence rates of NHL and its subtypes (aggressive and indolent) among Black Africans with and without HIV.

## Methods

### National Cancer Registry

We used data from the National Cancer Registry (NCR) in South Africa to identify incident NHL cases from 2010 to 2021. The NCR has been described in detail elsewhere.^13^ Briefly, the NCR is the legal entity mandated to collect and collate cancer data in South Africa. It was established in 1986 on a voluntary reporting basis. In April 2011, reporting of newly diagnosed cancers by health care providers became mandatory through Regulation 380 of the National Health Act of 2003. The NCR collects reports of histologically diagnosed cancers nation-wide and includes additional clinically diagnosed cancers at sentinel sites. Coders at the NCR, who classify these reports, are guided by the International Classification of Diseases for Oncology, 3rd edition (ICD-O-3).^14^ This analysis was limited to histologically confirmed cancers in the pathology-based registry where HIV status at cancer diagnosis is often not reported. Nonetheless the NCR uses natural language processing (NLP) and probabilistic record linkage (PRL) with other public health laboratory databases to assign HIV status to cancers. HIV status information is extracted from histopathology reports using NLP.^15^ For cancers without HIV status documented in the report, cancers are considered HIV negative if linked to a negative test within one year before diagnosis or at any time after diagnosis, and HIV positive if linked to a positive test before diagnosis or within one year after diagnosis.

### Estimated population size

We used data from various sources to estimate the annual Black African population count by HIV status. Briefly, we stratified the yearly South African population size by HIV status, sex, age group, population group (Black African, Coloured, Indian/Asian, White), and health care sector (private or public) by leveraging two data sources: the South African HIV Prevalence, Incidence, Behaviour and Communication Surveys (SABSSM) and the Thembisa model v4.6.^9,16^ From the SABSSM, we derived 1) distributions of population group and health care sector by sex, year and age group, and 2) relative risks of HIV positivity comparing people of different population groups and in different health care sectors, adjusting for sex, year, and age group. The Thembisa model provided population sizes by HIV status, sex, year, and age group. We provide a full explanation of the methodology used in these calculations in Supplementary Text 1.

### Inclusion criteria

We used ICD-O-3 morphology codes to identify NHL diagnoses reported to the NCR and grouped them by cell of origin and subtype as shown in Supplementary Table S1. This grouping follows the 5th edition of the World Health Organisation classification of haematolymphoid tumours.^17^ We limited our analysis to NHL diagnoses among Black Africans reported by public health laboratories, as private HIV-related laboratory data was unavailable. Only 10% of Black Africans in South Africa had medical insurance in 2023 with approximately 90% seeking care in the public sector.^18^ Thus, public health laboratory data are broadly representative of Black Africans in South Africa. In contrast, other population groups have higher private health insurance coverage, ranging from 20% among Coloured/Mixed race to 72% among White individuals in 2023.^18^ Because these population groups often seek care from the private sector,^18^ they tend to be under-represented in public health laboratory data and were, therefore, excluded from the current analysis.^18^

### Statistical analysis

We used descriptive statistics to characterise Black African individuals diagnosed with NHL by HIV status. Among NHL patients with known HIV status, we estimated HIV prevalence overall and by NHL subtypes. We used Poisson regression models with annual population size estimates as denominators, adjusted for sex, age and calendar period, to estimate adjusted incidence rate ratio (aIRR) for NHL and its subtypes comparing PWH and people without HIV (PWoH). We also calculated aIRR assessing the interaction between HIV status and calendar period (2010–2015 vs. 2016–−2021), sex (male vs. female), and age group (<45 [children, adolescents and young adults] vs. ≥45 years [middle-aged adults and elderly]). We used robust standard errors to account for overdispersion in the Poisson models. For the main regression analyses, we only included information from NHL patients with known HIV status. To assess the impact of missing HIV status on estimates we did a sensitivity analysis, where we tested how the extreme assumptions of considering all NHL patients with missing HIV status as either HIV negative or HIV positive affected our results.

### Ethics approval

We conducted this study using routinely collected data under the South African HIV Cancer Match study ethics approval (M240458) and the ethical waiver (W-CBP-230118–01) granted by the University of the Witwatersrand’s Human Research Ethics Committee. The waiver allows the NCR to fulfil its legislative mandate for cancer surveillance in South Africa and to support related research. It also permits researchers at the registry to publish epidemiological analyses based on routinely collected data without requiring individual informed consent or additional ethical approvals. Thus, we did not obtain individual patient consent for this analysis.

### Role of the funding source

The funders had no role in the analysis, interpretation, writing of the manuscript, or decision to submit findings for publication.

## Results

### Description of NHL cases

We identified 11,187 incident NHL diagnoses among Black Africans in public health facilities from 2010 to 2021. The median age at NHL diagnosis was 41 years (interquartile range [IQR]: 31–52), and 54% (n = 6023) occurred in male individuals (Table 1). About three-quarters of all NHL were B-cell lymphomas (73%, n = 8129), followed by NHL-not otherwise specified (NOS) (11%, n = 1238), precursor NHL (9%, n = 955), and T-cell lymphomas (8%, n = 865). Among B-cell lymphomas, DLBCL and BL were the most frequent subtypes, whereas for T-cell lymphomas, cutaneous T-cell lymphoma (CTCL) and PTCL, NOS were most common.

### NHL characteristics by HIV status

HIV status was known for 76% of incident NHL (n = 8526). The distribution of known HIV status varied across subtype with aggressive NHL subtypes having a higher proportion of known HIV status compared to indolent subtypes (Supplementary Table S2). The characteristics of individuals with missing HIV status are summarised in Table 1. Among those with a known HIV status, HIV prevalence was 81% (n = 6888) overall, ranging from 30% for CLL/SLL to 95% for CTCL (Fig. 1). While 26% of HIV-negative NHL patients were aged <15 years (n = 429), only 4% of HIV-positive NHL patients (n = 256) were in that age group (Table 1). In contrast, most HIV-positive NHL patients were aged 25–54 years (78%, n = 5362), and only 30% of HIV-negative NHL patients (n = 504) fell into that age category. Individuals aged ≥55 years, contributed to 32% of HIV-negative NHL and 12% of HIV-positive NHL. Supplementary Table S3 provides median age at diagnosis by HIV status for each NHL subtype. The proportion of male individuals was higher among HIV-negative (58%) than HIV-positive NHL patients (52%). DLBCL was the most common NHL subtype, irrespective of HIV status. However, its proportion was twice as high among HIV-positive NHL patients (61%; n = 4194) compared to HIV-negative patients (30%; n = 484). Among HIV-positive patients, DLBCL was followed by NHL-NOS (12%; n = 833) and BL (10%; n = 719). In HIV-negative patients, DLBCL was followed by precursor NHL (27%; n = 438) and CLL/SLL (16%; n = 254).

### Relative risk of NHL and its subtypes overall and by calendar period

Compared to PWoH, PWH had an elevated risk for all NHL (aIRR 24·2; 95% CI: 20·4–28·7) and each NHL subtype (Fig. 2). The aIRR was highest for CTCL (96·7; 95% CI: 50·6–184·9), followed by DLBCL (49·9; 95% CI: 38·5–64·8), and BL (48·8; 95% CI: 31·8–74·7). The aIRR was lowest for CLL/SLL (aIRR: 2·0; 95% CI: 1·3–3·2). For all NHL and most subtypes, the aIRRs were broadly similar between 2010–2015 and 2016–2021 (Fig. 3). Apparent aIRR declines were observed for BL (61·8 to 36·5), MZL (7·7 to 3·9), and ALCL (31·8 to 15·9). Conversely, aIRR tended to increase for PTCL-NOS (30·7 to 56·7), CTCL (72·1 to 174·8), and precursor NHL (2·6 to 3·9) from 2010 to 2015 to 2016–2021. However, confidence intervals overlapped across calendar periods.

### Relative risk of NHL and its subtypes by sex and age

For certain NHL subtypes, the relative risk among PWH compared to PWoH varied by sex and age. For all NHL and many subtypes, the aIRR tended to be higher in male than female individuals, but the p-values for interaction were >0·05 (Fig. 4). In contrast, the aIRR of other B-cell lymphomas was greater among female than male individuals. For all NHL, NHL-NOS, and most B-cell subtypes, except for BL, the aIRR was higher in individuals <45 years than those aged ≥45 years. Conversely, for precursor NHL, the aIRR was lower among younger individuals compared to older individuals (Fig. 5). For all T-cell subtypes and BL, the aIRR were similar across age groups.

### Sensitivity analyses

When we assumed that NHL patients with unknown HIV status were HIV negative in sensitivity analyses, most aIRR patterns remained similar to what we observed in the main analysis. We still found an elevated risk among PWH compared to PWoH for most NHL subtypes, except for CLL/SLL (aIRR: 0·9; 95% CI: 0·6–1·4) and other B-cell (aIRR: 1·7; 95% CI: 0·9–3·0) (Supplementary Fig. S1a). However, we no longer observed temporal changes in aIRR for BL and ALCL between 2010–2015 and 2016–2021. Furthermore, the aIRR for all NHL, PTCL-NOS, and CTCL now increased over calendar periods (Supplementary Fig. S2a). The sex and age patterns of the aIRR were similar in the main and this sensitivity analyses (Supplementary Figs. S3a and 4a). When we made the extreme assumption that NHL patients with missing HIV status were HIV positive, all aIRR patterns remained similar, but estimates were higher (Supplementary Figs. S1b to 4b).

## Discussion

Our study demonstrates that despite increasing ART access, Black Africans with HIV in South Africa remained at a substantially higher risk of NHL and its subtypes than those without HIV. The highest aIRR was observed for CTCL, followed by DLBCL and BL. The positive association with HIV was stronger among male individuals and those younger than 45 years for all NHL combined and many individual subtypes.

Our study is one of the first to examine the association of HIV with different NHL subtypes in a population with a high burden of HIV including both children and adults. The large number of NHL diagnoses over more than a decade allowed us to examine relative risks for many individual subtypes. We only included pathologically confirmed NHL diagnoses, eliminating uncertainties regarding NHL subtype in clinically diagnosed cases. Furthermore, we utilized PWoH as our reference group, as opposed to the general population used in other studies, avoiding a reduction in relative risks due to the contribution of PWH to the incidence rates in the general population. However, certain limitations must be considered. This study included only Black Africans, and the findings may not be generalizable to other population groups in South Africa. The missingness of HIV status varied between 13% and 48% across NHL subtypes with aggressive subtypes having a lower proportion of missingness compared to indolent subtypes. However, when we assumed that all NHL patients with unknown HIV status were HIV negative or HIV positive in sensitivity analyses, the observed patterns remained broadly similar for most NHL subtypes. T-cell NHL subtypes were rare, which reduced the precision of our estimates for these subtypes, especially after stratification by calendar period, sex, or age group. Also, we did not account for the additional uncertainty added by using model-based population denominators instead of actual population counts. Lastly, residual confounding may be present in our analysis as we were unable to adjust for other relevant factors such as lifestyle and environmental risk factors, family history, autoimmune diseases, ART status and immune/virological control among PWH, and co-infections with other oncogenic pathogens.

Our findings that Black Africans with HIV were more likely to be diagnosed with any NHL compared to those without HIV are consistent with findings from a large multi-country meta-analysis.^19^ This increased risk may be attributed to HIV-related immune modulation and greater susceptibility to infections, which contribute to the development of various NHL subtypes.^20^ HIV is known to induce immunosuppression and chronic immune activation that persists with ART.^3,4^ People with HIV also have a higher risk of acquiring and failing to control infections that contribute to NHL development such as Epstein–Barr virus, hepatitis B and C viruses, human herpesvirus 8, and human T-cell lymphotropic virus type 1.^6^ Furthermore, regular contact with health care systems may lead to higher diagnostic intensity and increase the likelihood of NHL diagnosis among PWH, and thus potentially also the observed strength of the association between HIV and NHL subtypes in our study. Nevertheless, under- and misdiagnosis of NHL remains a concern in South Africa, particularly among PWH, who are 10 times more likely to be on tuberculosis treatment at lymphoma diagnosis than PWoH.^21^ Additionally, one third of PWH die before an NHL diagnosis is made.^22^

Consistent with our findings, US-based studies reported an elevated relative risk among PWH for BL, DLBCL, NHL-NOS, MZL, ALCL, PTCL, and precursor NHL, with the strength of association varying across subtypes.^5,7^ This heterogeneity likely reflects differences in the role of immunosuppression, the contribution of oncogenic co-infections, and the distinct pathogenic pathways underlying each NHL subtype. For DLBCL, loss of immune control of the EBV seems to contribute to the increased risk among PWH.^23^ BL is less commonly associated with EBV and mostly driven by MYC translocation induced by HIV-1 related proteins that penetrate B-cells.^24^ Marginal zone lymphoma risk in PWH might be increased due to chronic antigenic stimulation by HIV or co-infection with hepatitis C virus, which is more prevalent among PWH than PWoH.^5^ HIV-associated depletion of CD4 T-cells could be associated with an increased PTCL risk, including PTCL-NOS, among PWH.^25^ The associations of ALCL and precursor NHL with HIV were substantially lower than those for DLBCL and BL, suggesting a less direct role of HIV in their pathogenesis, although the underlying mechanisms remain poorly understood.

Unlike our study, the population-based US studies did not show an association between HIV and FL, CLL/SLL, or CTCL.^5,7^ Additionally, a previous study in South Africa had found that PWH had a lower risk of FL than PWoH.^10^ The incidence rates of CLL/SLL and FL are known to increase with age.^26,27^ Infection with HIV leads to immune activation coupled with chronic inflammation, likely accelerating cellular aging in PWH,^4^ which may partly explain the associations between HIV and CLL/SLL or FL in our study. However, when we included all individuals with unknown HIV status as HIV negative, the association between HIV and CLL/SLL disappeared. Of note, CLL/SLL was the NHL subtype with the highest proportion of missing information on HIV status in our dataset (48%). The development of CTCL has been linked to chronic antigenic stimulation, which disrupts T-cell receptor signalling, leading to impaired immune surveillance and the proliferation of abnormal T-cells in the skin.^28^ HIV is known to interfere with T-cell signalling,^29^ which could explain the strong association of HIV and CTCL we observed. The discrepancies between our study and what was observed in the US might partly be attributed to the difference in the number of CTCL cases across studies. The estimated standardized incidence ratios for mycosis fungoides/Sezary syndrome in the US studies were based on 5 and 34 diagnoses only among PWH,^5,7^ whereas we included 241 individuals with CTCL and HIV, of whom 188 were diagnosed with mycosis fungoides.

The observed difference in the temporal pattern of aIRR by subtype suggest a complex interplay between immunosuppression, virological control, and aging in the pathogenesis of different NHL subtypes. Although ART coverage increased from 21% in 2010 to 72% in 2021 following the introduction of the universal test- and-treat policy in 2016,^9^ our study showed no evidence for changes in the aIRR for all NHL combined and most subtypes across calendar periods, except for BL, MZL, ALCL, PTCL-NOS, CTCL and precursor NHL. This may reflect limited changes in immunosuppression at ART initiation, despite ART coverage increasing over time. Supporting this, a study across five South African provinces found that CD4 cell counts at ART initiation, as recorded within the NHLS database, remained largely unchanged between 2011–2014 and 2015–2018.^30^ This could also explain the observed increase in PTCL-NOS in our study as PTCL in PWH has been associated with low CD4 cell counts and high HIV viral load.^25^ The weaker association of HIV and BL in the more recent calendar period is consistent with a previously reported decline in BL incidence among the Black African general population between 2012 and 2021 using data from the South African NCR.^31^ This suggests that the risk of BL among PWH in South Africa is decreasing with improved ART coverage. In South Africa, the HIV epidemic is generalized and predominantly affects female individuals, while in high-income countries HIV mostly affects male individuals who already have a higher BL risk than female individuals. Furthermore, many PWH in South Africa still start ART late with advanced HIV disease.^30,32^ The temporal increase in aIRR for precursor NHL that we observed does not align with stable or declining patterns reported in the US,^5,7^ and the reason for this difference warrants further investigations. In the US, compared with the general population, the risk of NHL and certain subtypes such as DLBCL was higher in the pre- and early-ART eras and steadily declined thereafter.^5,7,33^ In contrast, the relative risk for BL among PWH increased from the pre- to early-ART era and remained stable thereafter, with significant declines only starting in 2015–2019.^5,7,33^ For other subtypes, the strength of the association with HIV constantly declined over time.^7^ Whereas in 2015–2019, PWH continued to have a higher risk for DLBCL, BL, and PTCL than PWoH, risks were similar for FL and MZL and lower among PWH for CLL/SLL. The observed heterogeneity in temporal patterns may reflect differences in the HIV epidemics in both countries, including more sustained immune reconstitution in the US than in South Africa.^34,35^

In South Africa, ART coverage and immunosuppression vary by sex and age group. As of 2017, the proportion on ART was higher among female PWH aged 15 years or above (72%) compared to their male counterparts (68%), with the likelihood of being on ART increasing with age.^32^ Similarly, a greater proportion of female than male individuals on ART achieved viral suppression, with the proportion virally suppressed rising with older age until it declined again after the age of 50–64 years.^32^ These ART coverage patterns could partly explain why, for most NHL subtypes, we found an apparently stronger positive association with HIV among male individuals and those younger than 45 years.

In the era of widespread ART in South Africa, the incidence of all NHL and its subtypes remained elevated among Black Africans with HIV compared to those without HIV. Of note, the strength of the association with HIV for certain subtypes such as CTCL exceeded that of DLBCL and BL – two subtypes previously categorized as AIDS-defining. Our findings suggest that besides the well-known HIV-associated NHL subtypes, PWH are at increased risk of developing various other NHL subtypes. This may be attributed to improved survival rates among PWH and the accelerated aging process with persistent chronic inflammation and immune activation, despite effective viral suppression through ART.^3,4^ While ongoing efforts to manage chronic inflammation and immune activation are essential to address the increased risk of certain NHL subtypes in PWH, it is important for countries where HIV is endemic to develop strategies for the early detection of NHL in PWH to facilitate timely treatment and improve NHL outcomes. Improving NHL diagnosis is also required to adequately assess the burden of NHL associated with HIV. Furthermore, more NHL research from countries with a high HIV burden is needed as the characteristics of the HIV epidemic and NHL epidemiology may differ substantially from those in the US and Europe.

In conclusion, we found that, in South Africa’s public health sector, Black Africans with HIV had increased incidence rates of all NHL subtypes compared to Black Africans without HIV. Our findings underscore the need for countries with high HIV prevalence to implement strategies for the early detection of NHL in PWH, enabling timely treatment and improved outcomes after NHL diagnosis. More studies on NHL in countries with a high HIV burden are needed to better understand the relationship between HIV and individual NHL subtypes in these contexts.

## Supplementary Material

MMC1

## Figures and Tables

**Fig. 1:**
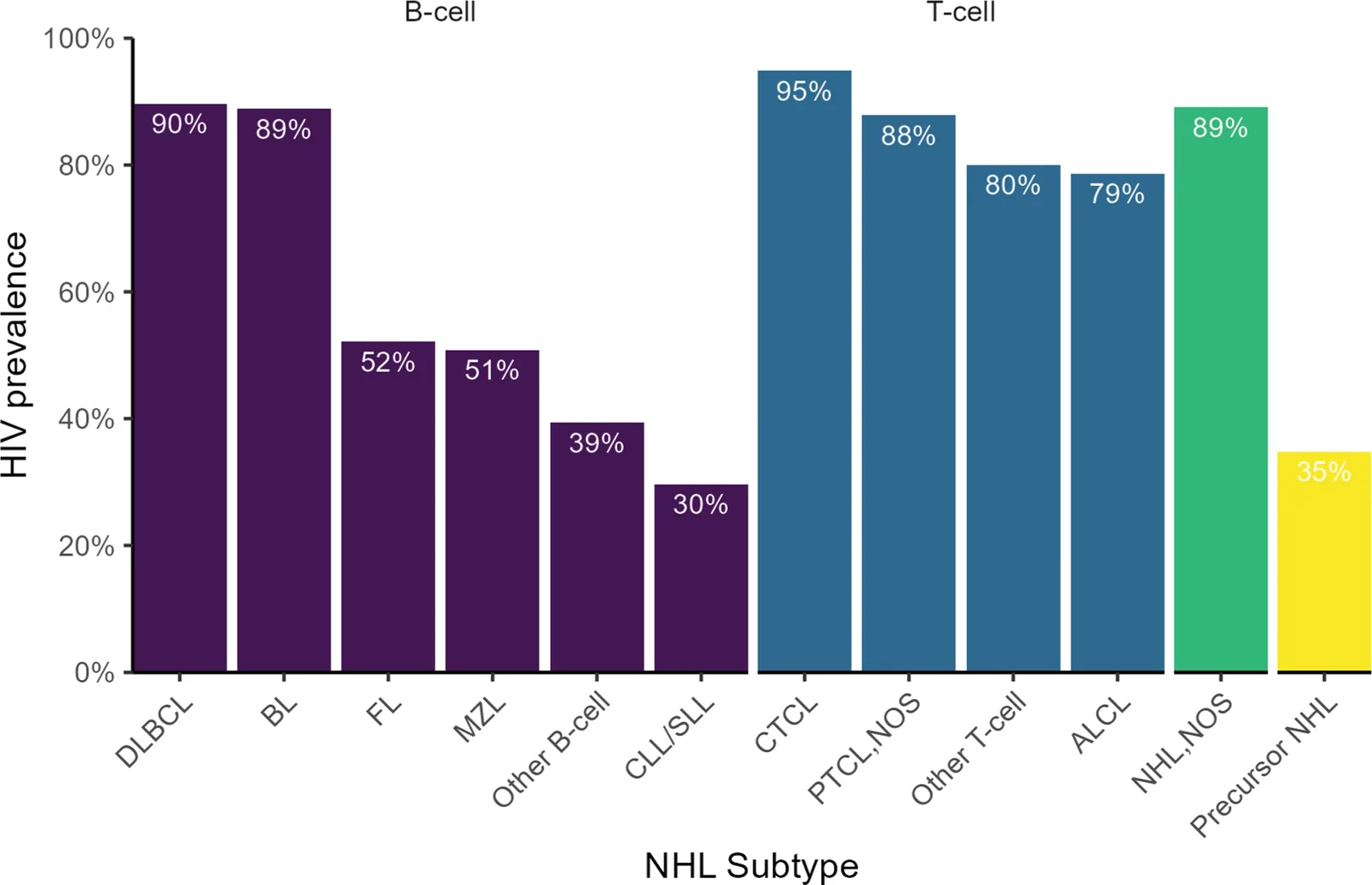
HIV prevalence by non-Hodgkin lymphoma subtype among individuals with a known HIV status. NHL: non-Hodgkin lymphoma; DLBCL: diffuse large B-cell lymphoma; BL: Burkitt lymphoma; FL: follicular lymphoma; CLL/SLL: chronic lymphocytic leukaemia/small lymphocytic lymphoma; MZL: marginal zone lymphoma; PTCL: peripheral T-cell lymphoma; NOS: not otherwise specified; ALCL: anaplastic large cell lymphoma; CTCL: cutaneous T-cell lymphoma.

**Fig. 2:**
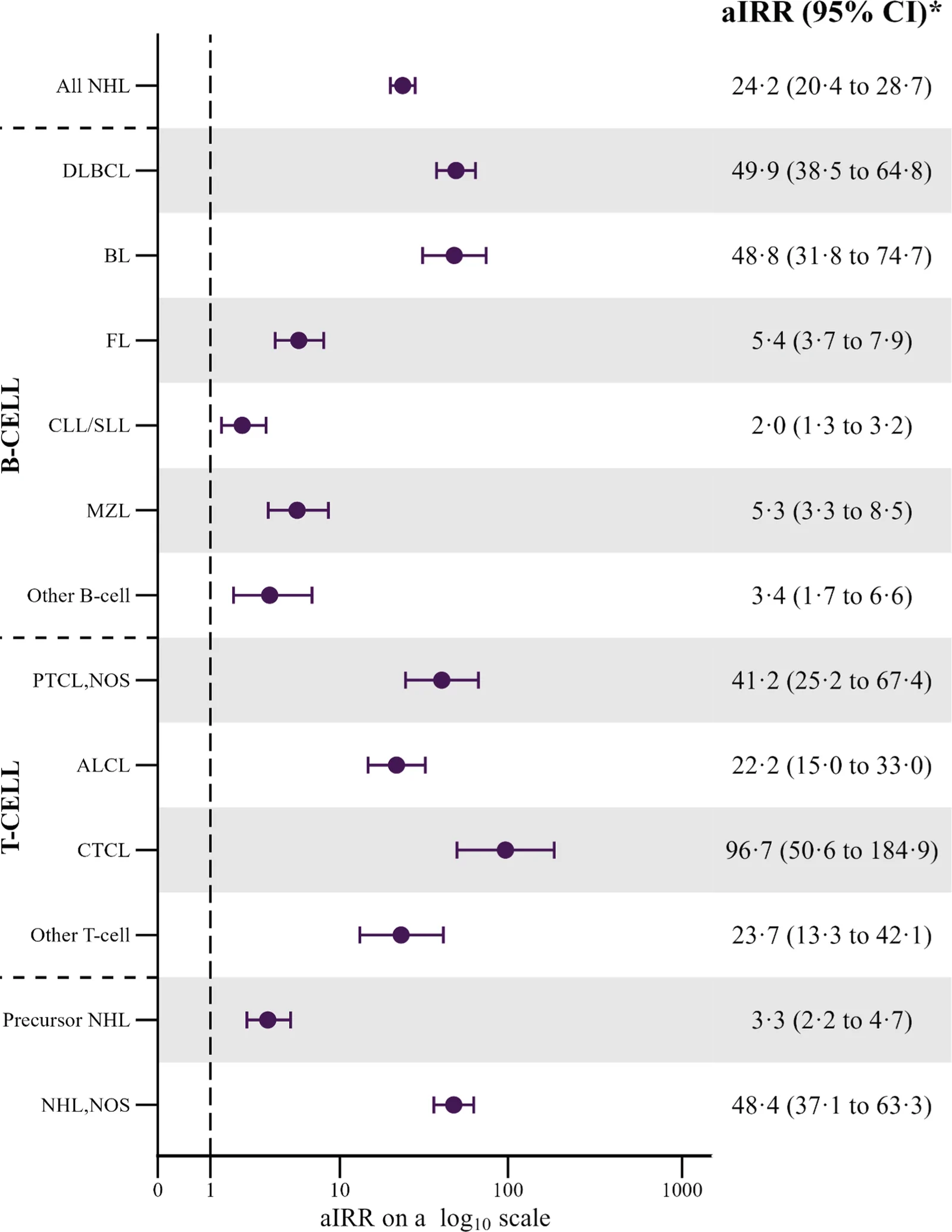
Overall adjusted incidence rate ratios of non-Hodgkin lymphoma subtypes among people with HIV compared to people without HIV. *The estimates are based on a model that adjusted for sex, age group, and calendar period. aIRR: adjusted incidence rate ratio; NHL: non-Hodgkin lymphoma; NOS: not otherwise specified; DLBCL: diffuse large B-cell lymphoma; BL: Burkitt lymphoma; FL: follicular lymphoma; CLL/SLL: chronic lymphocytic leukaemia/small lymphocytic lymphoma; MZL: marginal zone lymphoma; PTCL: peripheral T-cell lymphoma; ALCL: anaplastic large cell lymphoma; CTCL: cutaneous T-cell lymphoma.

**Fig. 3:**
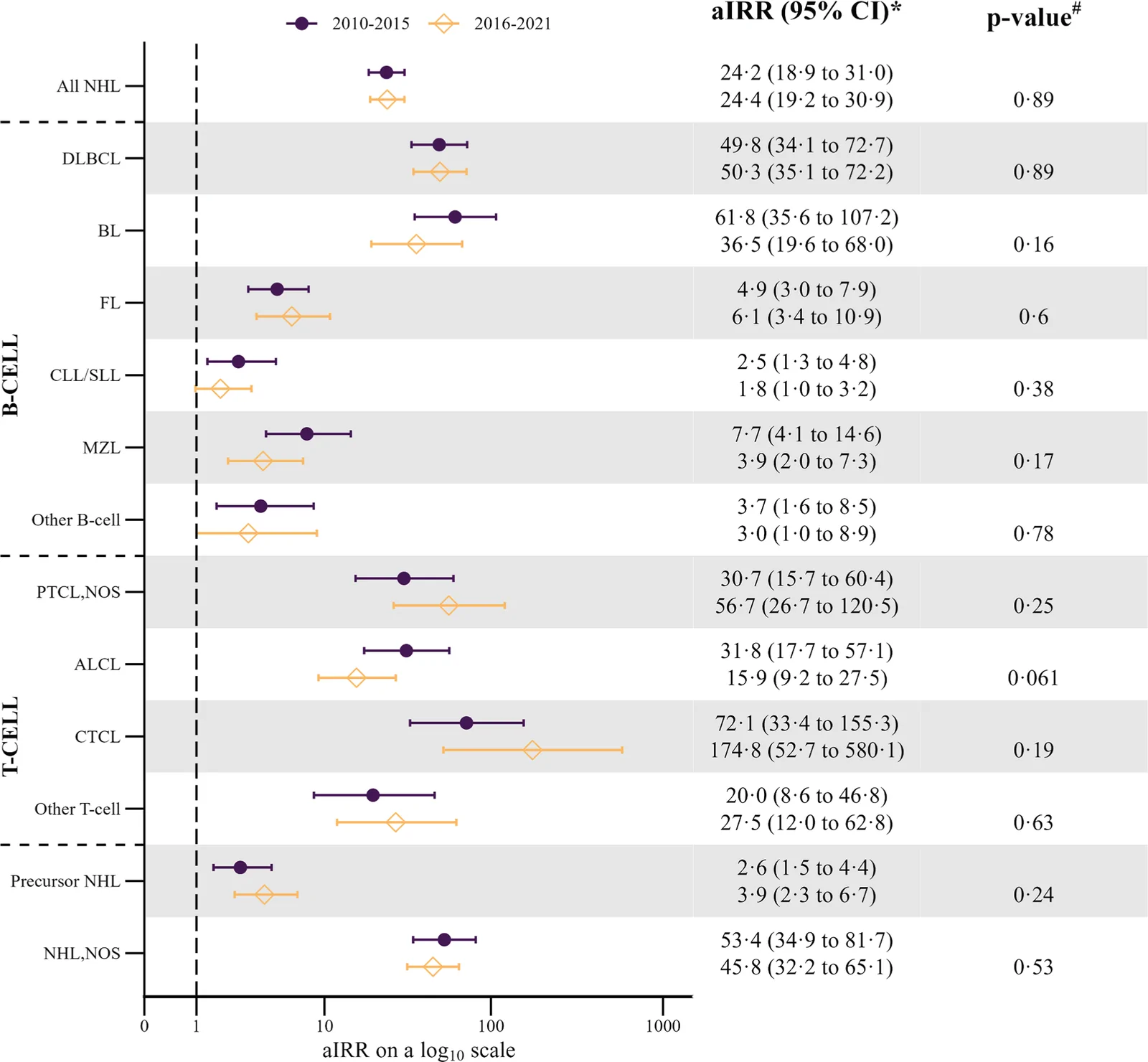
Adjusted incidence rate ratios of non-Hodgkin lymphoma subtypes by calendar period among people with HIV compared to people without HIV. *The estimates are based on a model that adjusted for sex and age group. #The p-values are testing for an interaction between calendar year and HIV status. aIRR: adjusted incidence rate ratio; NHL: non-Hodgkin lymphoma; DLBCL: diffuse large B-cell lymphoma; BL: Burkitt lymphoma; FL: follicular lymphoma; CLL/SLL: chronic lymphocytic leukaemia/small lymphocytic lymphoma; MZL: marginal zone lymphoma; PTCL: peripheral T-cell lymphoma; NOS: not otherwise specified; ALCL: anaplastic large cell lymphoma; CTCL: cutaneous T-cell lymphoma.

**Fig. 4:**
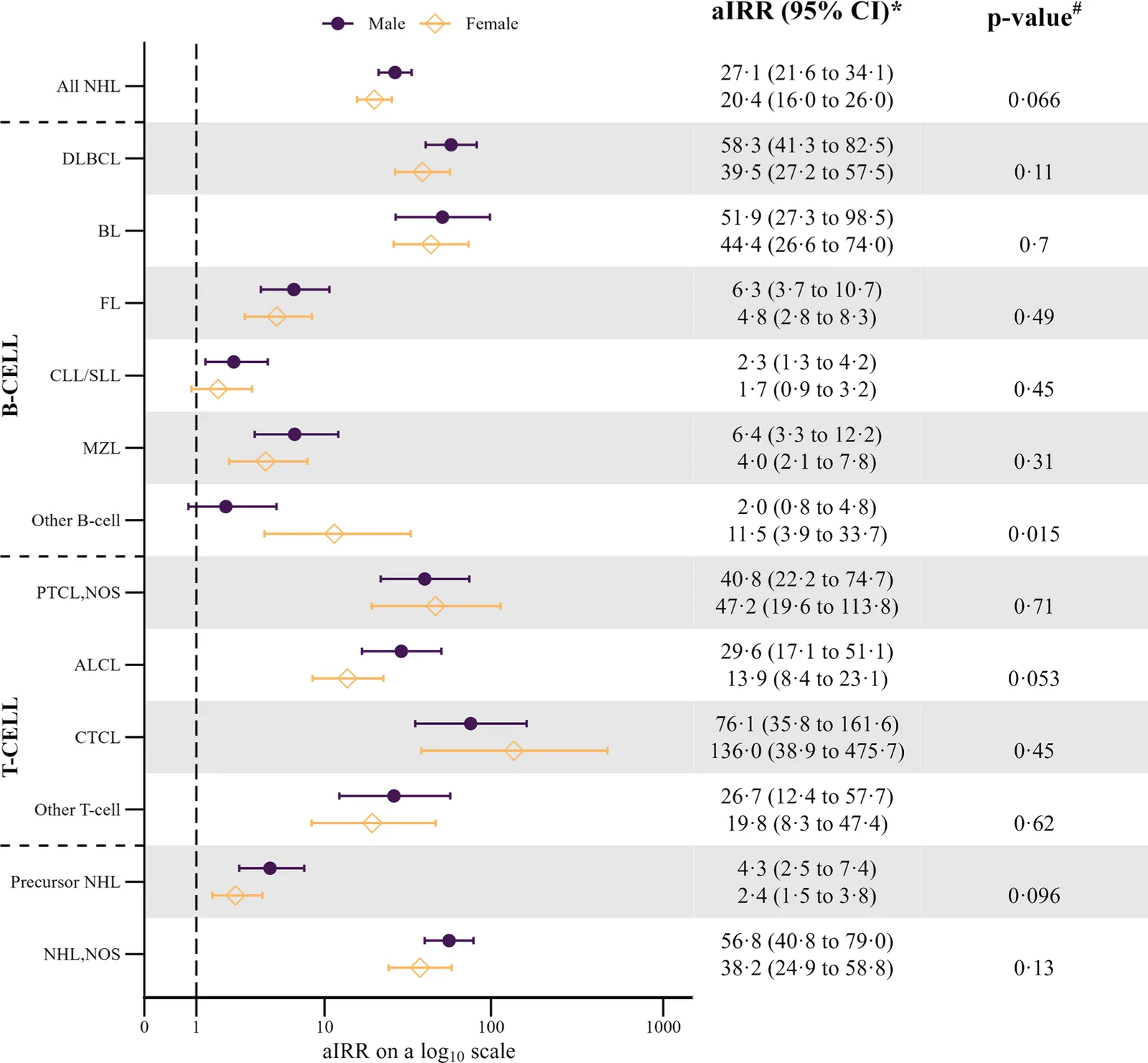
Adjusted incidence rate ratios of non-Hodgkin lymphoma subtypes by sex among people with HIV compared to people without HIV. *The estimates are based on a model that adjusted for age group and calendar period. #The p-values are testing for an interaction between sex and HIV status. aIRR: adjusted incidence rate ratio; NHL: non-Hodgkin lymphoma; DLBCL: diffuse large B-cell lymphoma; BL: Burkitt lymphoma; FL: follicular lymphoma; CLL/SLL: chronic lymphocytic leukaemia/small lymphocytic lymphoma; MZL: marginal zone lymphoma; PTCL: peripheral T-cell lymphoma; NOS: not otherwise specified; ALCL: anaplastic large cell lymphoma; CTCL: cutaneous T-cell lymphoma.

**Fig. 5:**
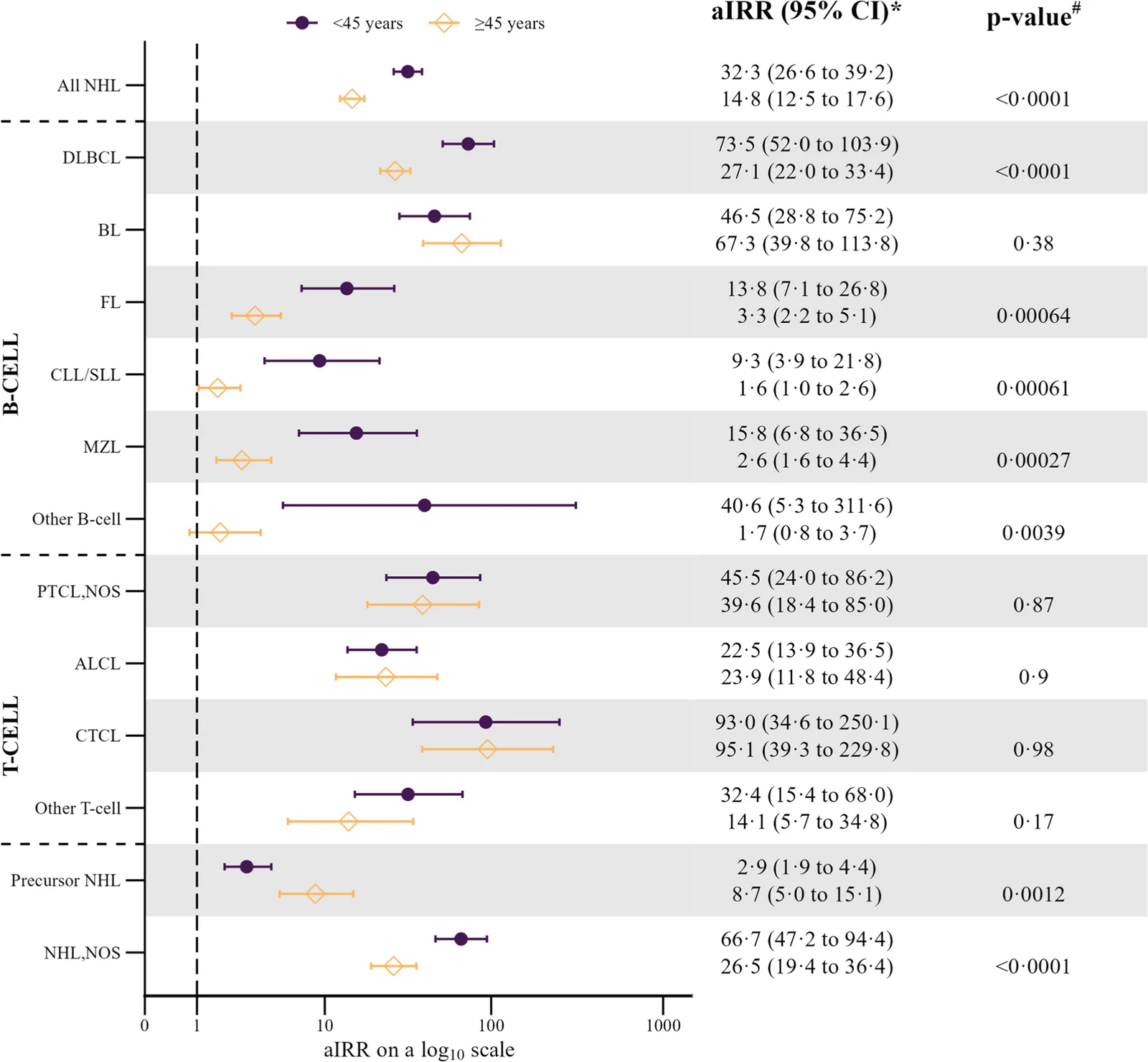
Adjusted incidence rate ratios of non-Hodgkin lymphoma subtypes by age group among people with HIV compared to people without HIV. *The estimates are based on a model that adjusted for sex and calendar period. #The p-values are testing for an interaction between age group and HIV status. aIRR: adjusted incidence rate ratio; NHL: non-Hodgkin lymphoma; DLBCL: diffuse large B-cell lymphoma; BL: Burkitt lymphoma; FL: follicular lymphoma; CLL/SLL: chronic lymphocytic leukaemia/small lymphocytic lymphoma; MZL: marginal zone lymphoma; PTCL: peripheral T-cell lymphoma; NOS: not otherwise specified; ALCL: anaplastic large cell lymphoma; CTCL: cutaneous T-cell lymphoma.

**Table 1:** Characteristics of individuals with a non-Hodgkin lymphoma diagnosis by HIV status.

	HIV-negative	HIV-positive	HIV-unknown	Total
	(N = 1638)	(N = 6888)	(N = 2661)	(N = 11,187)
Median age (Q1, Q3)	38 (13, 58)	40 (33, 48)	45 (31, 60)	41 (31, 52)
Age group (years)				
<15	429 (26%)	256 (4%)	277 (11%)	962 (9%)
15–24	176 (11%)	369 (5%)	152 (6%)	697 (6%)
25–34	169 (10%)	1414 (21%)	342 (14%)	1925 (18%)
35–44	131 (8%)	2423 (35%)	489 (19%)	3043 (28%)
45–54	204 (12%)	1525 (22%)	391 (16%)	2120 (19%)
55–64	274 (17%)	625 (9%)	392 (16%)	1291 (12%)
≥65	249 (15%)	216 (3%)	478 (19%)	943 (9%)
Missing	6	60	140	206
Sex				
Female	688 (42%)	3308 (48%)	1145 (43%)	5141 (46%)
Male	948 (58%)	3569 (52%)	1506 (57%)	6023 (54%)
Missing	2	11	10	23
Calendar period				
2010–2015	770 (47%)	3128 (45%)	1566 (59%)	5464 (49%)
2016–2021	868 (53%)	3760 (55%)	1095 (41%)	5723 (51%)
NHL type				
B-cell	**1004 (61%)**	**5191 (75%)**	**1934 (73%)**	**8129 (73%)**
DLBCL	484 (30%)	4194 (61%)	1246 (47%)	5924 (53%)
BL	90 (5%)	719 (10%)	158 (6%)	967 (9%)
FL	78 (5%)	85 (1%)	74 (3%)	237 (2%)
CLL/SLL	254 (16%)	107 (2%)	338 (13%)	699 (6%)
MZL	58 (4%)	60 (1%)	77 (3%)	195 (2%)
Other B-cell	40 (2%)	26 (0%)	41 (2%)	107 (1%)
Precursor NHL	**438 (27%)**	**234 (3%)**	**283 (11%)**	**955 (9%)**
T-cell	**94 (6%)**	**630 (9%)**	**141 (5%)**	**865 (8%)**
PTCL,NOS	24 (1%)	174 (3%)	45 (2%)	243 (2%)
CTCL	41 (3%)	151 (2%)	43 (2%)	235 (2%)
ALCL	13 (1%)	241 (3%)	39 (1%)	293 (3%)
Other T-cell	16 (1%)	64 (1%)	14 (1%)	94 (1%)
NOS	**102 (6%)**	**833 (12%)**	**303 (11%)**	**1238 (11%)**

NHL, non-Hodgkin lymphoma; DLBCL, diffuse large B-cell lymphoma; BL, Burkitt lymphoma; FL, follicular lymphoma; CLL/SLL, chronic lymphocytic leukaemia/small lymphocytic lymphoma; MZL, marginal zone lymphoma; PTCL, peripheral T-cell lymphoma; NOS, not otherwise specified; ALCL, anaplastic large cell lymphoma; CTCL, cutaneous T-cell lymphoma.

## Data Availability

The National Cancer Registry data supporting the findings of this study are available upon reasonable request through the National Health Laboratory Service Academic Affairs and Research Management System (https://aarms.nhls.ac.za). The South African HIV Prevalence, Incidence, Behaviour and Communication Surveys data can be requested from Human Sciences Research Council Research Data Service (https://hsrc-repository.figshare.com/) and the Thembisa estimates are openly available at https://www.thembisa.org/downloads.

## Research in context

### Evidence before this study

We searched PubMed for articles with combinations of the terms “HIV”, “immunodeficiency”, “lymphoma”, “non-Hodgkin”, “B-cell”, “diffuse large B cell”, “Burkitt”, “T-cell”, “primary effusion”, “primary central nervous system”, “follicular”, “marginal zone”, “chronic lymphocytic leukaemia”, “small lymphocytic lymphoma”, “acute lymphoblastic leukaemia”, “anaplastic large cell”, “high grade”, “low grade”, “epidemiology”, “incidence” and “Africa” with no restriction on publication date at the inception of the study in June 2025. We also examined the reference lists of selected publications to identify articles about non-Hodgkin lymphoma (NHL) and its subtypes among people with HIV (PWH). We found a study in the United States (US) that compared the risk of NHL and its subtypes between PWH and the general population, with an updated analysis published in August 2025. In these studies, HIV was strongly associated with aggressive NHL subtypes but had moderate to no association with indolent NHL, with the strength of these associations declining over time. In sub-Saharan Africa, existing studies were hospital-based and focused on HIV’s association with all NHL combined, or aggressive NHL subtypes only.

### Added value of this study

To our knowledge, this is the first study to compare incidence rates of NHL subtypes between PWH and people without HIV (PWoH) at a national level in a country with a high HIV prevalence. We found that among South Africa’s Black African population, PWH had a substantially higher risk of developing any NHL subtype than PWoH with no evidence of changes in relative risk across calendar periods, except for Burkitt lymphoma (BL), peripheral T-cell lymphoma, not otherwise specified (PTCL-NOS), and precursor NHL. The strongest association with HIV was observed for cutaneous T-cell lymphomas, diffuse large B cell lymphoma (DLBCL), and BL. The association with HIV tended to be stronger among male individuals and those aged <45 years for all NHL combined and many subtypes.

### Implications of all the available evidence

Our findings suggest that besides the well-known HIV-associated NHL subtypes such as DLBCL and BL, PWH are at increased risk of developing various other NHL subtypes. This may be attributed to improved survival among PWH and the accelerated aging process with persistent chronic inflammation despite effective viral suppression through antiretroviral therapy. More NHL research from countries in sub-Saharan Africa with a high HIV burden is needed as the characteristics of the HIV epidemics and possibly also the NHL epidemiology in this region are distinct from those in the US and Europe. Meanwhile, it is important for countries where HIV is endemic to develop strategies for the early detection of NHL in PWH to facilitate timely treatment and improve NHL outcomes.

## Appendix A. Supplementary data

Supplementary data related to this article can be found at https://doi.org/10.1016/j.lanafr.2026.100058.
