# Supplementary material for: Incidence rates of non-Hodgkin lymphoma subtypes among Black Africans with and without HIV in South Africa: a national registry-based study
Source: Lancet Reg Health Afr. Author manuscript; Available in PMC 2026 Oct 10. (PMC13652135; doi:10.1016/j.lanafr.2026.100058)
Supplement: Supplementary file 1 [file NIHMS2174500-supplement-MMC1.pdf]

Incidence rates of non-Hodgkin lymphoma subtypes among Black Africans with and without HIV in South Africa: a national registry-based study

SUPPLEMENTARY MATERIALS

Table of content

Supplementary Text 1: Methodology used to calculation population estimates 2

Supplementary Table 1: Non-Hodgkin lymphoma International Classification of Diseases for Oncology, 3rd edition morphology codes by subtype 4
Supplementary Table 2: Proportion of non-Hodgkin lymphoma subtypes with known and missing HIV status ... 4
Supplementary Table 3: Median age at diagnosis of non-Hodgkin lymphoma subtypes by HIV status 4

Supplementary Figure 1: Overall adjusted incidence rate ratios of non-Hodgkin lymphoma subtypes among people with HIV compared to people without HIV with two-sided extreme assumptions 5
Supplementary Figure 2: Adjusted incidence rate ratios of non-Hodgkin lymphoma subtype by calendar period among people with HIV with two-sided extreme assumptions... 6
Supplementary Figure 3:Adjusted incidence rate ratios of non-Hodgkin lymphoma subtype by sex among people with HIV compared to people without HIV with two-sided extreme assumptions.. 7
Supplementary Figure 4: Adjusted incidence rate ratios of non-Hodgkin lymphoma subtype by age group among people with HIV compared to people without HIV with two-sided extreme assumptions 8

Supplementary Text 1: Methodology used to calculation population estimates

We calculated population estimates by sex, age group, population group (Black African, Coloured, Indian/Asian, White), health sector (private vs public) and year using data from the South African HIV Prevalence, Incidence, Behaviour and Communication Surveys (SABSSM) and Thembisa model v4.6. The SABSSM is a cross-sectional survey that is carried out every few years in South Africa (2002, 2005, 2008, 2012, 2017). It aims to identify the HIV status of South Africans and the presence of social and behavioural risk factors for HIV. The surveys gather individual-level information on a participant's age, sex, population group, laboratory confirmed HIV-status, and where they usually obtain health care services. We classified age into 5-year age groups (0-4, 5-9, …) up to age 80, and a single group for those aged 80 years or older. Based on a person's answer to the question "*Where do you usually obtain health care?"*, we defined the individual as accessing care either in the public sector (public hospital, public clinic or doctor, government hospital, day hospital/clinic community health centre, mobile clinic, family planning clinic) or the private sector (private hospital, private clinic or doctor, pharmacy, miner hospital, traditional healer, other private medical).

The Thembisa model provides estimates on the population living with HIV in South Africa stratified by sex, age, and calendar year. We stratified these estimates further by population group and health sector, by leveraging the results of the SABSSM using the following decomposition (Formula 1):

$$\begin{aligned}
&Nb\ of\ HIV\ positive\ people\ of\ age\ x, sex\ g, race\ r, in\ sector\ s\ and\ in\ year\ y \\
&= (Nb\ of\ people\ of\ age\ x, sex\ g\ in\ year\ y) \\
&* (Proportion\ of\ people\ of\ age\ x, sex\ g, in\ year\ y\ who\ are\ HIV\ positive, of\ race\ r, and\ in\ sector\ s) \\
&= \big(N_0(g,x,y) + N_1(g,x,y)\big) * P(HIV\ positive, r, s \mid g,x,y) \\
&= \big(N_0(g,x,y) + N_1(g,x,y)\big) * P(r,s \mid g,x,y) * P(HIV\ positive \mid r,s,g,x,y),
\end{aligned}$$

[Formula 1]

where $N_0(g,x,y)$ and $N_1(g,x,y)$ are respectively the Thembisa estimates of the HIV-negative and HIV-positive South African population of sex g and age x in year y.

Similarly, we estimated the HIV negative population with (Formula 2):

$$Nb\ of\ HIV\ negative\ people\ of\ age\ x, sex\ g, race\ r, in\ sector\ s\ and\ in\ year\ y = \big(N_0(g,x,y) + N_1(g,x,y)\big) * P(r,s \mid g,x,y) * (1 - P(HIV\ positive \mid r,s,g,x,y)).$$

[Formula 2]

The quantity $P(r,s \mid g,x,y)$ found in Formulas 1 and 2 denotes the proportion of people of sex g and age x in year y who are in a population group r and in sector s. We derived this from the SABSSM surveys for $y =$ 2008, 2012, and 2017, incorporating the weights provided with the survey for analysing questionnaire data. We excluded individuals with missing weights when estimating this proportion. We used linear interpolation to extend this proportion to the years in between surveys and carried forward the proportion from 2017 to 2018-2021.

The HIV prevalence $P(HIV\ positive \mid r,s,g,x,y)$ in Formulas 1 and 2 was estimated as (Formula 3).

$$P(HIV\ positive \mid r = Black\ African, s = Public, g,x,y) * R(r,s),$$

[Formula 3]

where $R(r,s)$ is the risk ratio for HIV comparing someone of population group r and in sector s to a Black African in the public sector of the same sex, the same age, and in the same year. We estimated these ratios by fitting a weighted generalized linear model to the SABSSM data, with HIV status as binomial response and a log-link function. We used the weights provided by the SABSSM surveys for the analysis of specimen data. Individuals with a missing weight or no information on sector (public/private) were excluded from these regressions.

We calculated the quantity $P(HIV\ positive \mid r = Black\ African, s = Public, g,x,y)$ found in Formula 3 using the following identity:

$$\begin{aligned}
N_1(g,x,y) &= \big(N_0(g,x,y) + N_1(g,x,y)\big) * P(HIV\ positive \mid g,x,y) \\
&= \big(N_0(g,x,y) + N_1(g,x,y)\big) * \sum_r \sum_s P(r,s \mid g,x,y) * P(HIV\ positive \mid r,s,g,x,y),
\end{aligned}$$

and thus,

$$P(HIV\ positive \mid r = Black\ African, s = Public, g, x, y) = \frac{N_1(g,x,t)}{\big(N_0(g,x,t) + N_1(g,x,t)\big) \sum_r \sum_s P(r, s \mid g, x, y)\, R(r,m)},$$

with $R(r = Black\ African, s = Public) = 1$.

Supplementary Table 1: Non-Hodgkin lymphoma International Classification of Diseases for Oncology, 3rd edition morphology codes by subtype

Cell of origin	Subtype	International Classification of Diseases for Oncology, 3rd edition morphology code
B-Cell	Diffuse large B-cell lymphoma	9678/3, 9679/3, 9680/3, 9684/3, 9688/3, 9735/3, 9737/3, 9738/3
	Burkitt lymphoma	9687/3, 9826/3
	Follicular lymphoma	9690/3, 9691/3, 9695/3, 9698/3
	Chronic lymphocytic leukaemia / small lymphocytic lymphoma	9670/3, 9823/3
	Marginal zone B-cell lymphoma	9689/3, 9699/3, 9764/3
	Other B-cell lymphomas	9597/3, 9761/3, 9671/3, 9673/3, 9833/3, 9940/3,
T-Cell	Peripheral T-cell lymphoma, not otherwise specified	9702/3,
	Anaplastic large cell lymphoma	97143
	Cutaneous T-cell lymphoma	9700/3, 9708/3, 9709/3, 9718/3
	Other T-cell lymphomas	9701/3, 9705/3, 9716/3, 9717/3, 9719/3, 9827/3, 9834/3, 9948/3
-	Acute lymphoblastic leukaemia / lymphomas	9727/3, 9728/3, 9729/3, 9811/3, 9812/3, 9835/3, 9836/3, 9837/3
-	Non-Hodgkin, not otherwise specified	9591/3, 9832/3, 9820/3, 9596/3

Supplementary Table 2: Proportion of non-Hodgkin lymphoma subtypes with known and missing HIV status

Subtype	Known HIV-status	Missing/Unknown HIV-status
DLBCL	79%	21%
BL	84%	16%
FL	69%	31%
CLL/SLL	52%	48%
MZL	61%	39%
Other B-cell	62%	38%
PTCL, NOS	81%	19%
ALCL	82%	18%
CTCL	87%	13%
Other T-cell	85%	15%
Precursor NHL	70%	30%
NHL, NOS	76%	24%

DLBCL: diffuse large B-cell lymphoma; BL: Burkitt lymphoma; FL: follicular lymphoma; CLL/SLL: chronic lymphocytic leukaemia/small lymphocytic lymphoma; MZL: marginal zone lymphoma; PTCL: peripheral T-cell lymphoma; NOS: not otherwise specified; ALCL: anaplastic large cell lymphoma; CTCL: cutaneous T-cell lymphoma

Supplementary Table 3: Median age at diagnosis of non-Hodgkin lymphoma subtypes by HIV status

Subtype	HIV-negative Median age (IQR)	HIV-positive Median age (IQR)	HIV-unknown Median age (IQR)
DLBCL	44.5 (28;58.5)	40 (34;47)	43 (34;55)
BL	6 (4;13)	36 (29;43)	32 (15;44)
FL	54 (46;61)	46 (35;52)	60 (47;67)
CLL/SLL	61.5 (54;69)	55 (45;62)	66 (57;74)
MZL	53.5 (45;66)	43 (35;53)	59 (42.5;68)
Other B	60 (54;69)	48 (37;57)	63 (48;69)
PTCL, NOS	30.5 (26;60)	42.5 (32;57.5)	43 (29;63)
ALCL	16 (8;42)	39 (29;48)	41 (16;58)
CTCL	48 (40;56)	50 (38;61.5)	53 (43;70)
other T	40 (27;56.5)	40.5 (32.5;50)	46.5 (32;60)
Precursor	10 (5;19)	25 (13;40)	11 (5;22)
NHL, NOS	41.5 (19.5;58)	40 (33;47)	45 (32;57)

IQR: Interquartile range; DLBCL: diffuse large B-cell lymphoma; BL: Burkitt lymphoma; FL: follicular lymphoma; CLL/SLL: chronic lymphocytic leukaemia/small lymphocytic lymphoma; MZL: marginal zone lymphoma; PTCL: peripheral T-cell lymphoma; NOS: not otherwise specified; ALCL: anaplastic large cell lymphoma; CTCL: cutaneous T-cell lymphoma

Supplementary Figure 1: Overall adjusted incidence rate ratios of non-Hodgkin lymphoma subtypes among people with HIV compared to people without HIV with two-sided extreme assumptions

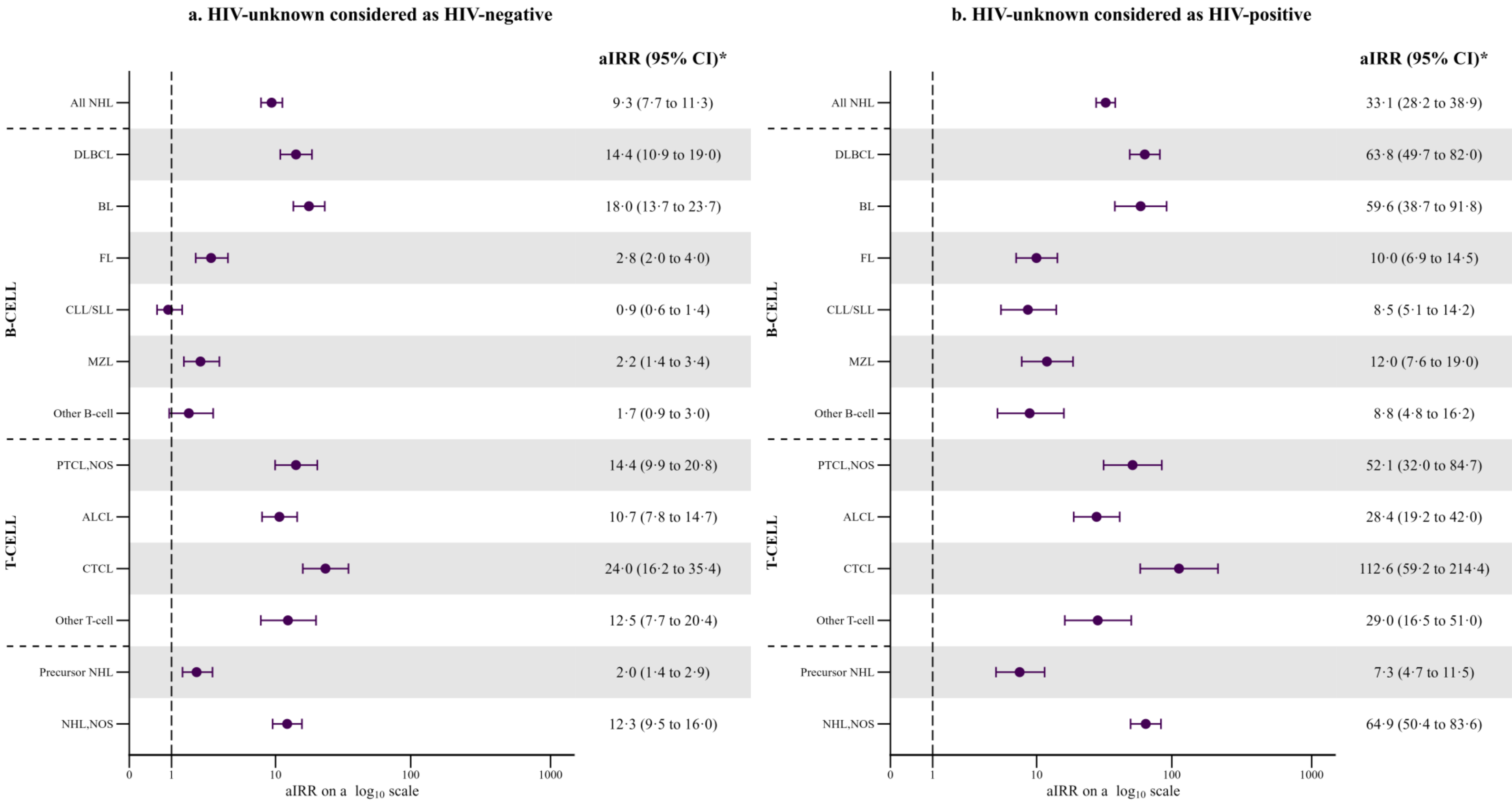

*The estimates are based on a model that adjusted for sex, age group, and calendar period. aIRR: adjusted incidence rate ratio; NHL: non-Hodgkin lymphoma; DLBCL: diffuse large B-cell lymphoma; BL: Burkitt lymphoma; FL: follicular lymphoma; CLL/SLL: chronic lymphocytic leukaemia/small lymphocytic lymphoma; MZL: marginal zone lymphoma; PTCL: peripheral T-cell lymphoma; NOS: not otherwise specified; ALCL: anaplastic large cell lymphoma; CTCL: cutaneous T-cell lymphoma

Supplementary Figure 2: Adjusted incidence rate ratios of non-Hodgkin lymphoma subtype by calendar period among people with HIV with two-sided extreme assumptions

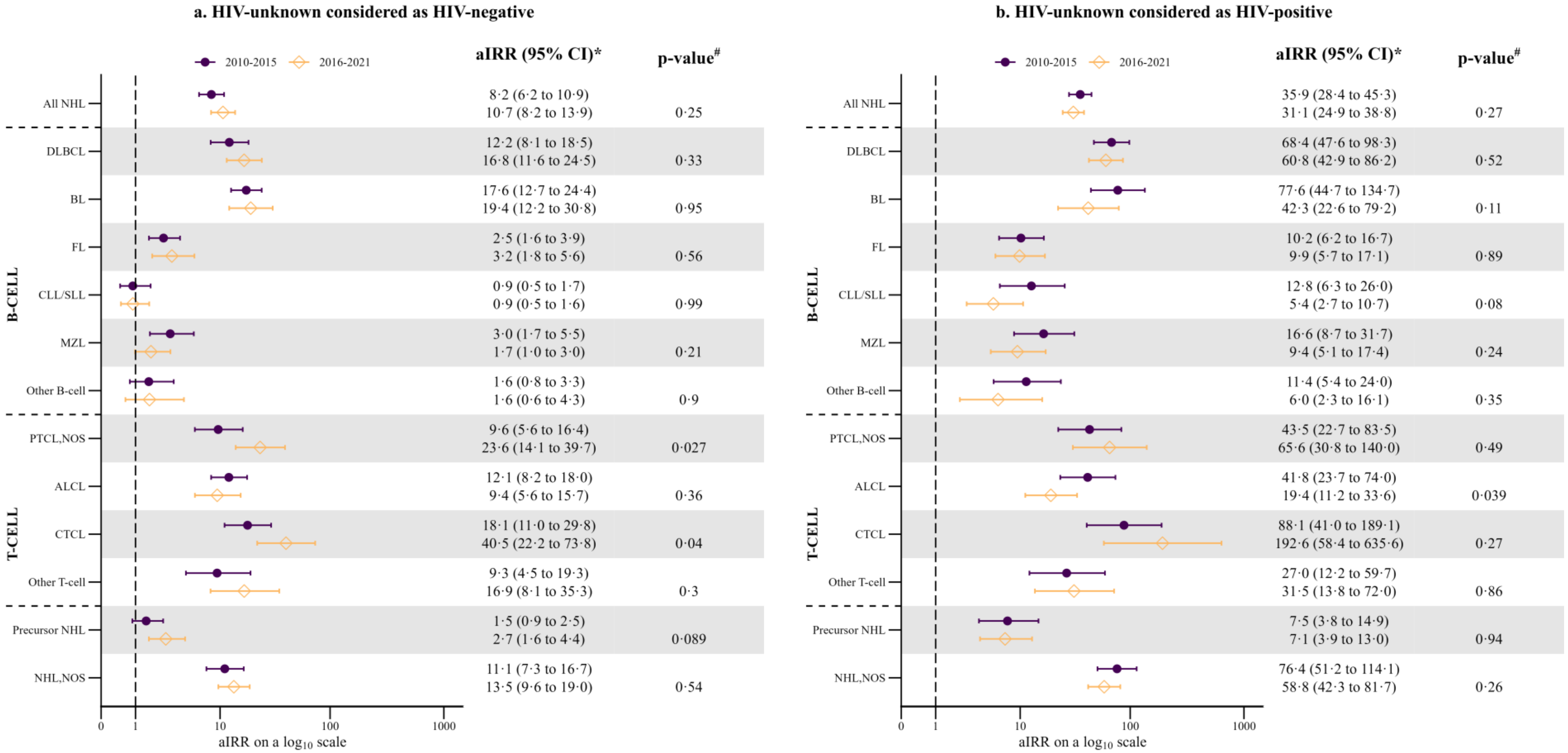

*The estimates are based on a model that adjusted for sex and age group.

#The p-values are testing for an interaction between calendar year and HIV status.

aIRR: adjusted incidence rate ratio; NHL: non-Hodgkin lymphoma; DLBCL: diffuse large B-cell lymphoma; BL: Burkitt lymphoma; FL: follicular lymphoma; CLL/SLL: chronic lymphocytic leukaemia/small lymphocytic lymphoma; MZL: marginal zone lymphoma; PTCL: peripheral T-cell lymphoma; NOS: not otherwise specified; ALCL: anaplastic large cell lymphoma; CTCL: cutaneous T-cell lymphoma

Supplementary Figure 3:Adjusted incidence rate ratios of non-Hodgkin lymphoma subtype by sex among people with HIV compared to people without HIV with two-sided extreme assumptions

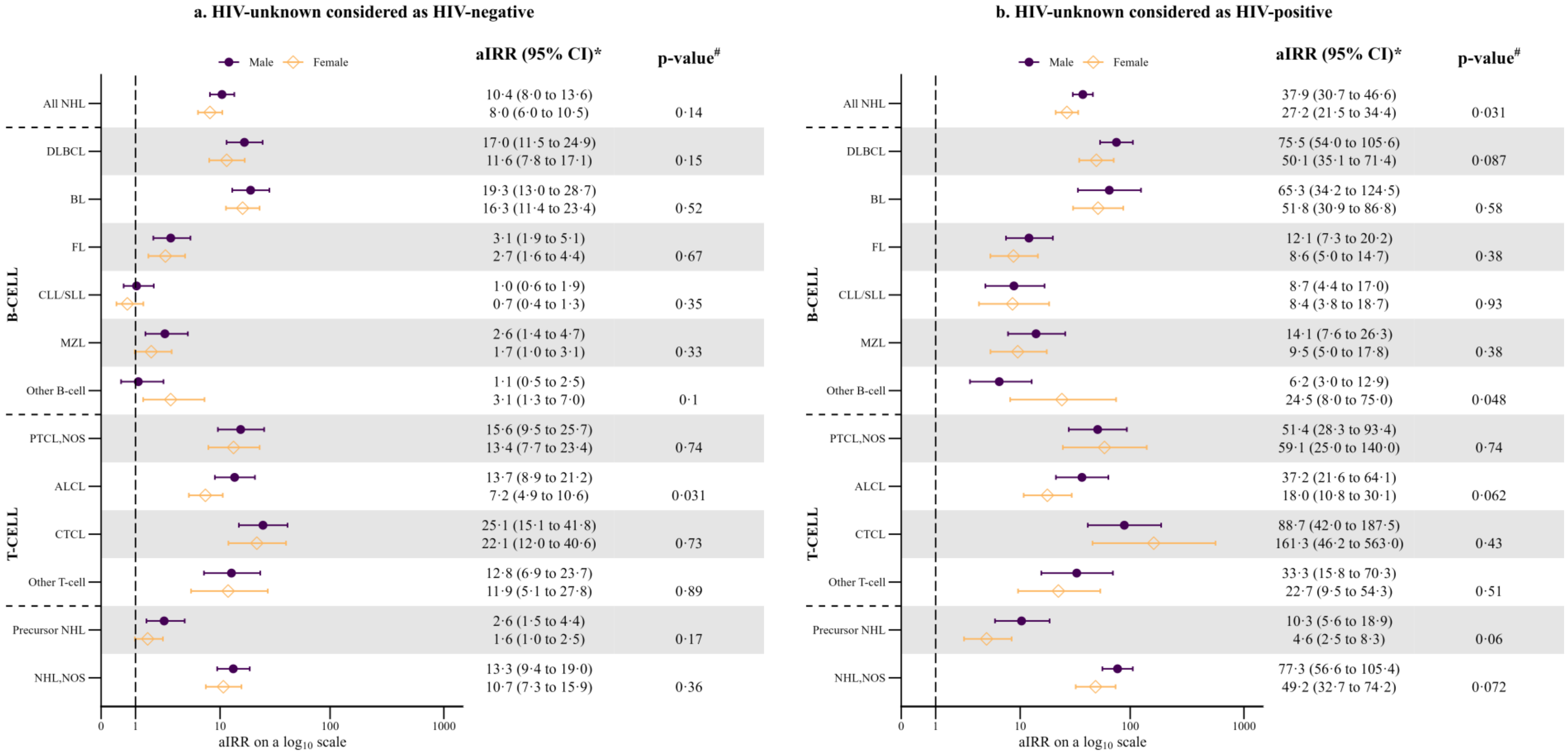

*The estimates are based on a model that adjusted for age group and calendar period.

#The p-values are testing for an interaction between sex and HIV status.

aIRR: adjusted incidence rate ratio; NHL: non-Hodgkin lymphoma; DLBCL: diffuse large B-cell lymphoma; BL: Burkitt lymphoma; FL: follicular lymphoma; CLL/SLL: chronic lymphocytic leukaemia/small lymphocytic lymphoma; MZL: marginal zone lymphoma; PTCL: peripheral T-cell lymphoma; NOS: not otherwise specified; ALCL: anaplastic large cell lymphoma; CTCL: cutaneous T-cell lymphoma

Supplementary Figure 4: Adjusted incidence rate ratios of non-Hodgkin lymphoma subtype by age group among people with HIV compared to people without HIV with two-sided extreme assumptions

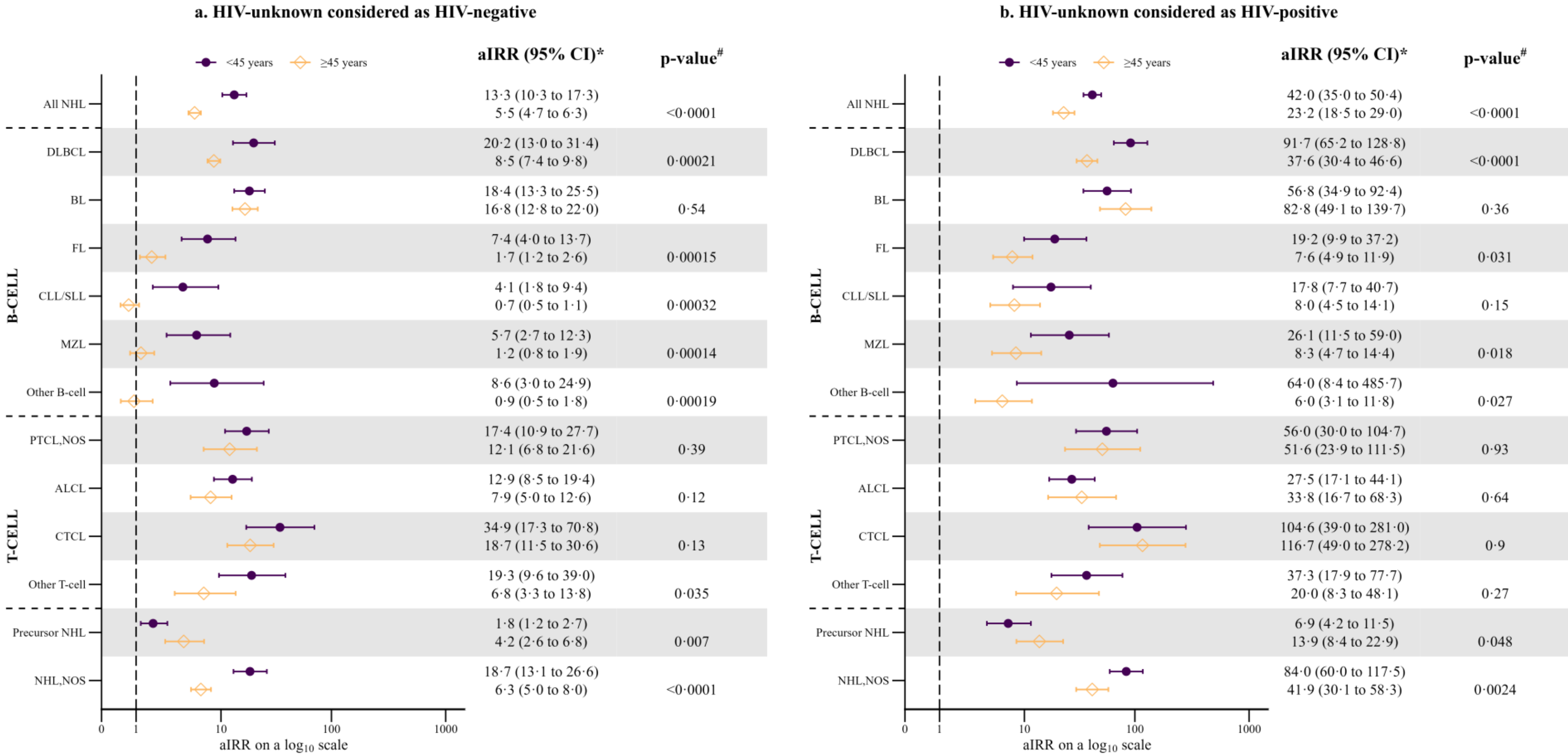

*The estimates are based on a model that adjusted for sex and calendar period.

#The p-values are testing for an interaction between age group and HIV status.

aIRR: adjusted incidence rate ratio; NHL: non-Hodgkin lymphoma; DLBCL: diffuse large B-cell lymphoma; BL: Burkitt lymphoma; FL: follicular lymphoma; CLL/SLL: chronic lymphocytic leukaemia/small lymphocytic lymphoma; MZL: marginal zone lymphoma; PTCL: peripheral T-cell lymphoma; NOS: not otherwise specified; ALCL: anaplastic large cell lymphoma; CTCL: cutaneous T-cell lymphoma
